# A deep learning model based on chest CT to predict benign and malignant breast masses and axillary lymph node metastasis

**DOI:** 10.17305/bb.2025.12010

**Published:** 2025-03-17

**Authors:** Jingxiang Sun, Xiaoming Xi, Mengying Wang, Menghan Liu, Xiaodong Zhang, Haiyan Qiu, Youxin Zhang, Taian Fu, Yanan Du, Wanqing Ren, Dawei Wang, Guang Zhang

**Affiliations:** 1 Department of Radiology, The First Affiliated Hospital of Shandong First Medical University & Shandong Provincial Qianfoshan Hospital, Shandong, China; 2 Postgraduate Department, Shandong First Medical University (Shandong Academy of Medical Sciences), Shandong, China; 3 School of Computer Science and Technology, Shandong Jianzhu University, Shandong, China; 4 Department of Postgraduate, Shandong Second Medical University, Shandong, China; 5 Department of Health Management, The First Affiliated Hospital of Shandong First Medical University & Shandong Provincial Qianfoshan Hospital, Shandong, China; 6 Department of Radiology, Jinan Third People’s Hospital, Shandong, China; 7 Shandong Engineering Research Center of Health Management, Jinan, China; 8 Shandong Institute of Health Management, Jinan, China

**Keywords:** Axillary lymph node metastasis, ALNM, breast cancer, breast mass, chest CT, deep learning

## Abstract

Differentiating early-stage breast cancer from benign breast masses is crucial for radiologists. Additionally, accurately assessing axillary lymph node metastasis (ALNM) plays a significant role in clinical management and prognosis for breast cancer patients. Chest computed tomography (CT) is a commonly used imaging modality in physical and preoperative evaluations. This study aims to develop a deep learning model based on chest CT imaging to improve the preliminary assessment of breast lesions, potentially reducing the need for costly follow-up procedures such as magnetic resonance imaging (MRI) or positron emission tomography-CT and alleviating the financial and emotional burden on patients. We retrospectively collected chest CT images from 482 patients with breast masses, classifying them as benign (*n* ═ 224) or malignant (*n* ═ 258) based on pathological findings. The malignant group was further categorized into ALNM-positive (*n* ═ 91) and ALNM-negative (*n* ═ 167) subgroups. Patients were randomly divided into training, validation, and test sets in an 8:1:1 ratio, with the test set excluded from model development. All patients underwent non-contrast chest CT before surgery. After preprocessing the images through cropping, scaling, and standardization, we applied ResNet-34, ResNet-50, and ResNet-101 architectures to differentiate between benign and malignant masses and to assess ALNM. Model performance was evaluated using sensitivity, specificity, accuracy, receiver operating characteristic (ROC) curves, and the area under the curve (AUC). The ResNet models effectively distinguished benign from malignant masses, with ResNet-101 achieving the highest performance (AUC: 0.964; 95% CI: 0.948–0.981). It also demonstrated excellent predictive capability for ALNM (AUC: 0.951; 95% CI: 0.926–0.975). In conclusion, these deep learning models show strong diagnostic potential for both breast mass classification and ALNM prediction, offering a valuable tool for improving clinical decision-making.

## Introduction

Breast cancer is the most prevalent malignancy among women worldwide and remains the fifth leading cause of cancer-related mortality in this population [1]. The American Cancer Society estimates that by 2025, nearly 320,000 individuals in the United States will be diagnosed with breast cancer, with more than 42,000 expected to succumb to the disease [2]. Early diagnosis and timely intervention are critical for improving breast cancer prognosis [3, 4], underscoring the need for effective screening and diagnostic strategies. Another key factor influencing prognosis is the evaluation of axillary lymph node (ALN) involvement. An accurate assessment of ALN metastasis (ALNM) helps clinicians determine disease stage, select appropriate surgical interventions, and develop postoperative adjuvant treatment plans [5, 6]. Conventional breast imaging methods include mammography, ultrasound (US), magnetic resonance imaging (MRI), and positron emission tomography-computed tomography (PET-CT). However, these techniques have certain limitations, such as patient discomfort, difficulty in obtaining definitive diagnoses with a single modality, high costs, and prolonged examination times. With the increasing prevalence of physical examinations and standardized hospital protocols—especially after the COVID-19 pandemic—the number of chest computed tomography (CT) scans has risen significantly, covering a broad range of clinical indications. Although chest CT is not a routine screening tool for breast lesions, its imaging scope typically includes the entire breast region. Several studies have explored the potential of utilizing this incidental imaging data for breast cancer diagnosis [7, 8]. As a result, chest CT is becoming an increasingly viable first-line diagnostic tool for detecting new breast lesions [9, 10]. In chest CT images of advanced breast cancer, features such as irregular tumor edges, asymmetric shape, skin thickening, lymph node enlargement, and chest wall or skin invasion are often prominent. In contrast, early-stage breast cancer presents with subtler characteristics, making it more challenging to differentiate from benign masses with the naked eye. Thus, distinguishing early breast cancer from benign lesions is crucial for improving patient outcomes. Additionally, prior research has demonstrated that tumor characteristics—such as morphology, density or signal intensity, and margin definition—can serve as predictive indicators of ALNM [11, 12]. Therefore, in this study, we aimed to develop a noninvasive and accurate method to predict ALNM status using chest CT imaging data.

In recent years, the rapid advancement of artificial intelligence (AI) has positioned AI-assisted diagnostic imaging as a key research focus, significantly transforming the field of medical imaging. Unlike traditional machine learning methods, which require manual feature extraction, deep learning autonomously learns features directly from data using artificial neural network architectures. In computer vision tasks, convolutional neural networks (CNNs) have demonstrated remarkable efficacy. Several clinical applications of CNNs have been proposed, particularly in radiology, where they have been extensively studied for classification, detection, and segmentation [13]. Koh et al. [14] evaluated the performance of a RetinaNet-based network in detecting breast cancer on contrast-enhanced chest CT images. Their results indicated that the deep learning algorithm could sensitively identify breast cancer on CT scans. However, the study lacked a benign control group, raising concerns about the robustness and generalizability of the findings. Zhang et al. [15] and Yang et al. [16] developed and validated deep learning models for predicting ALNM in breast cancer patients using multi-phase CT images. These studies employed rigorous methodologies, and their models demonstrated strong predictive accuracy (ACC). While deep learning algorithms based on chest CT images show promise for improving early breast cancer diagnosis and ALNM prediction [14–16], most prior studies have focused on contrast-enhanced CT images. The use of contrast agents, however, carries potential risks such as allergic reactions and nephrotoxicity. Additionally, existing models often predict either the benign/malignant nature of breast masses or ALNM, with relatively few capable of addressing both simultaneously. Our study aims to develop a robust deep learning model using non-contrast chest CT images to predict both the benign or malignant nature of breast masses and the likelihood of ALNM. By leveraging non-contrast CT images, we seek to facilitate preliminary diagnosis and risk assessment during routine physical examinations or initial hospital visits. This approach eliminates the need for contrast agents, reduces reliance on additional imaging modalities like MRI or PET-CT, and ultimately saves examination time and costs while minimizing unnecessary radiation exposure.

## Materials and methods

This retrospective study received approval from the institutional review board, and the requirement to obtain informed consent was waived.

**Figure 1.**
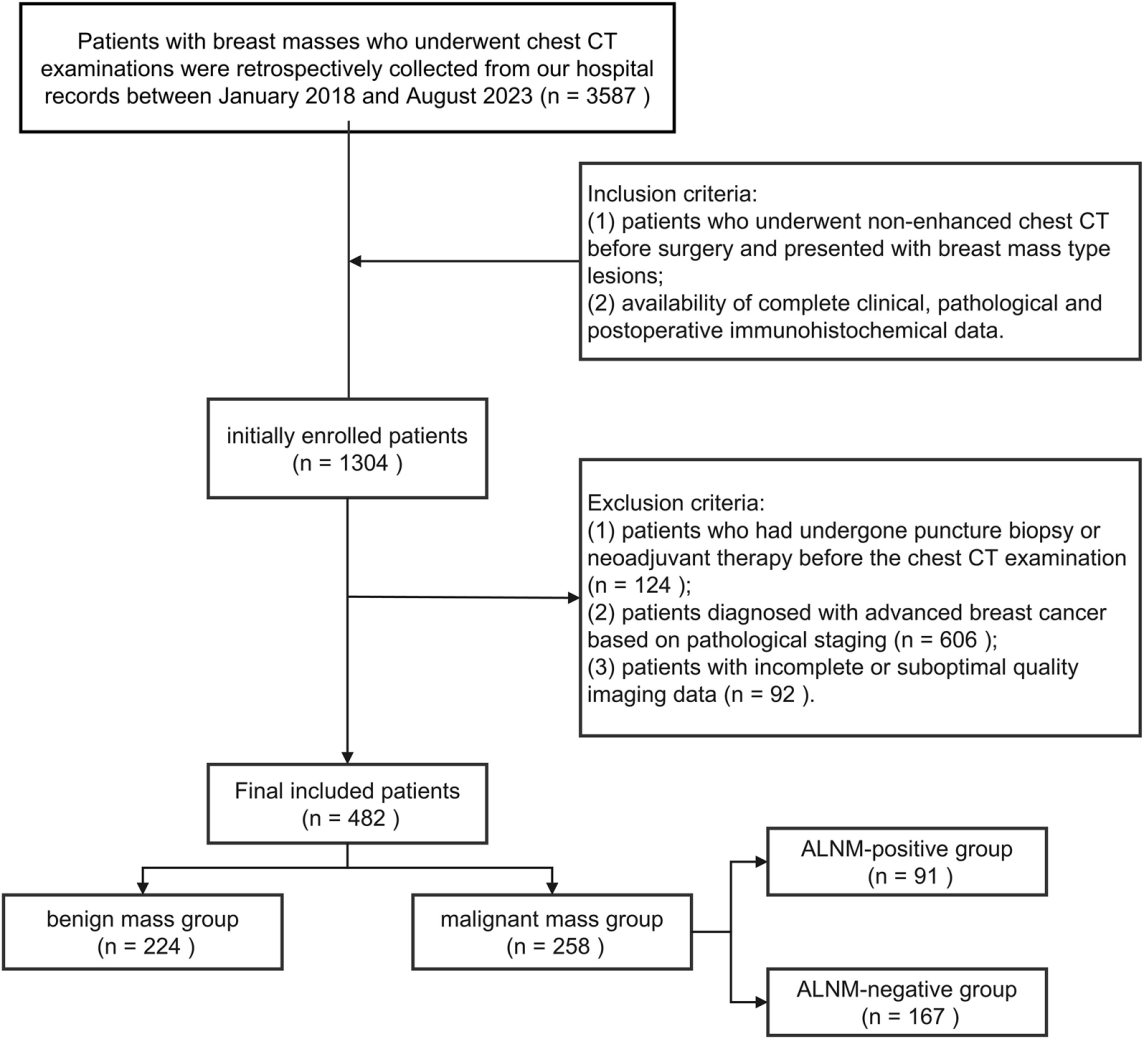
**The enrollment process of patients with breast masses.** Abbreviations: ALNM: Axillary lymph node metastasis; CT: Computed tomography.

### Population

This study retrospectively collected clinicopathological and imaging data from patients with breast masses who underwent non-contrast-enhanced chest CT scans before surgery at the First Affiliated Hospital of Shandong First Medical University between January 2018 and August 2023. Based on pathological findings, patients were categorized into benign and malignant mass groups. The benign mass group included: ① Fibroadenoma ② Intraductal papilloma ③ Other benign lesions (e.g., phyllodes tumors, breast cysts, hamartomas). The malignant mass group consisted exclusively of early-stage non-specific invasive carcinoma. According to the eighth edition of the TNM staging system and American Joint Committee on Cancer (AJCC) clinical staging guidelines, early-stage breast cancer is defined as T0-2N0-1M0, indicating a tumor diameter of ≤5 cm with ALNM limited to ≤3 nodes. Based on postoperative pathological confirmation of ALN status, the malignant group was further divided into: ALNM-positive (pathological stage ≥ pN1a), ALNM-negative (pathological stage pN0). Inclusion criteria: Patients who underwent non-contrast chest CT before surgery with documented breast mass lesions; availability of complete clinical, pathological, and postoperative immunohistochemical data. Exclusion criteria: Patients who had a prior biopsy or neoadjuvant therapy before CT; patients diagnosed with advanced breast cancer based on pathological staging; patients with incomplete or poor-quality imaging data. The inclusion and exclusion process is illustrated in Figure 1. Ultimately, 482 patients were enrolled, comprising 224 benign and 258 malignant cases. Among the malignant cases, 91 were ALNM-positive and 167 were ALNM-negative. Baseline characteristics for both groups are summarized in Table 1, with further details on the ALNM subgroups in Table 2. Patients were randomly allocated into training (*n* ═ 386), validation (*n* ═ 48), and test (*n* ═ 48) sets in an 8:1:1 ratio, with the test set excluded from algorithm development.

### CT imaging parameters

Non-contrast chest CT images were acquired using various CT scanners, including the Insurance CT (Philips), uCT550 (United Shadow), Revolution 256 (GE Healthcare), uCT960+ (United Shadow), and Discovery CT750 HD (GE Healthcare). Patients were positioned supine with arms raised. The scans extended from the thoracic inlet to the posterior costophrenic angle and were performed at 120 kV with variable tube currents ranging from 100 to 300 mA. Other scanning parameters included a pitch of 0.8–1.0, a matrix of 512 × 512, a slice thickness of 1–1.25 mm, a window width of 350 Hounsfield units (HU), and a window level of +40 HU. To ensure comprehensive visualization of the entire breast region and prevent any omission of breast mass information, axial thin-section mediastinal window images were selected for analysis.

**Table 1:** Baseline characteristics between benign and malignant groups

**Characteristics**	**Total**	**Benign cohort**	**Malignant cohort**	***P* value**
Number	482	224	258	
Age, year	49 (36.75–59)	37 (26–49.75)	57 (48–67)	<0.001
Tumor size, cm	2.3 (1.8–3.0)	2.3 (1.6–3.2)	2.3 (1.9–3.0)	0.589

Continuous variables with skewed distributions are presented as median and quartile.

**Table 2:** Baseline characteristics between ALNM-positive and ALNM-negative groups

**Characteristics**	**ALNM-positive**	**ALNM-negative**	***P* value**
Number	91	167	
Age, years old	55.99 ± 13.11	57.69 ± 12.02	0.293
Tumor size, cm	2.52 ± 0.80	2.43 ± 0.85	0.406
Histologic grade (%)			0.143
I	6 (6.6)	11 (6.6)	
II	60 (65.9)	90 (53.9)	
III	25 (27.5)	66 (39.5)	
Molecular subtype (%)			0.080
Luminal A	46 (50.5)	62 (37.1)	
Luminal B	28 (30.8)	55 (32.9)	
HER2-positive	9 (9.9)	18 (10.8)	
TN	8 (8.8)	32 (19.2)	

Continuous variables are described as mean ± standard deviation, and categorical variables are presented as numbers (%). Abbreviations: ALNM: Axillary lymph node metastasis; TN: Triple-negative.

### Image processing

**Figure 2.**
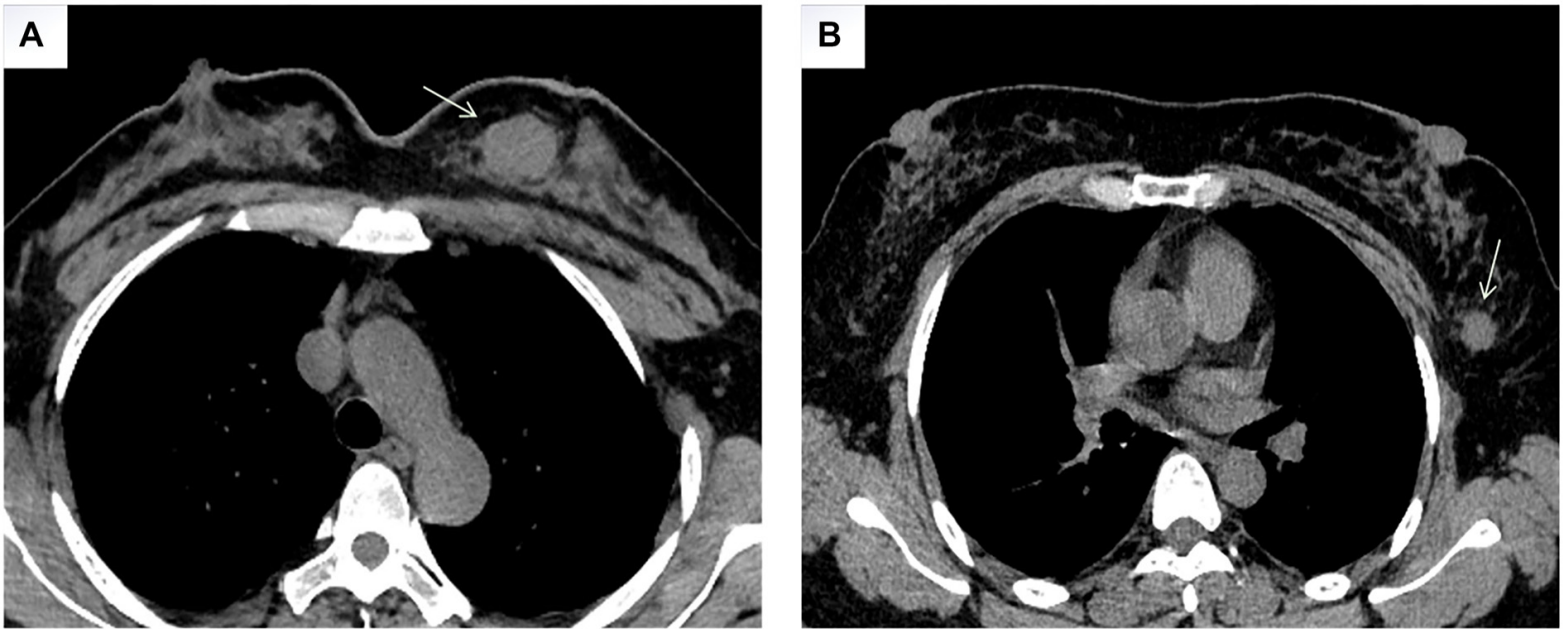
**An example of chest CT images that completely include breast tissue.** Arrows point to the locations of breast masses. (A) Represents a benign breast mass (breast fibroadenoma); (B) Denotes a malignant breast mass (early non-specific invasive breast cancer). Abbreviation: CT: Computed tomography.

First, diagnostic radiologists re-evaluated the collected chest CT images and delineated the region fully covering the breast tissue (Figure 2). This process was carried out by two doctors with five years of experience in breast imaging. In cases of disagreement, a senior doctor with 15 years of experience provided an independent judgment. Subsequently, all re-framed images were resized to 224×224 pixels. Finally, image normalization was applied using Equation (1) to scale pixel values within the range [0, 1]. 
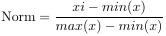

where *xi* represents the pixel value, while *max*(*x*) and *min*(*x*) represent the maximum and minimum values of the image pixels, respectively.

**Table 3:** The learning parameters of each ResNet model

	**ResNet 34**	**ResNet 50**	**ResNet 101**
Epoch	100	100	100
Batch size	16	16	16
Ir	0.001^*#^	0.0001^*^/0.001^#^	0.001^*#^
Optimizer	Adam	Adam	Adam

^*^Refers to the parameter of the Resnet models for predicting benign and malignant breast masses; ^#^Presents the parameter of the ALNM model.

### Development of deep learning models

**Figure 3.**
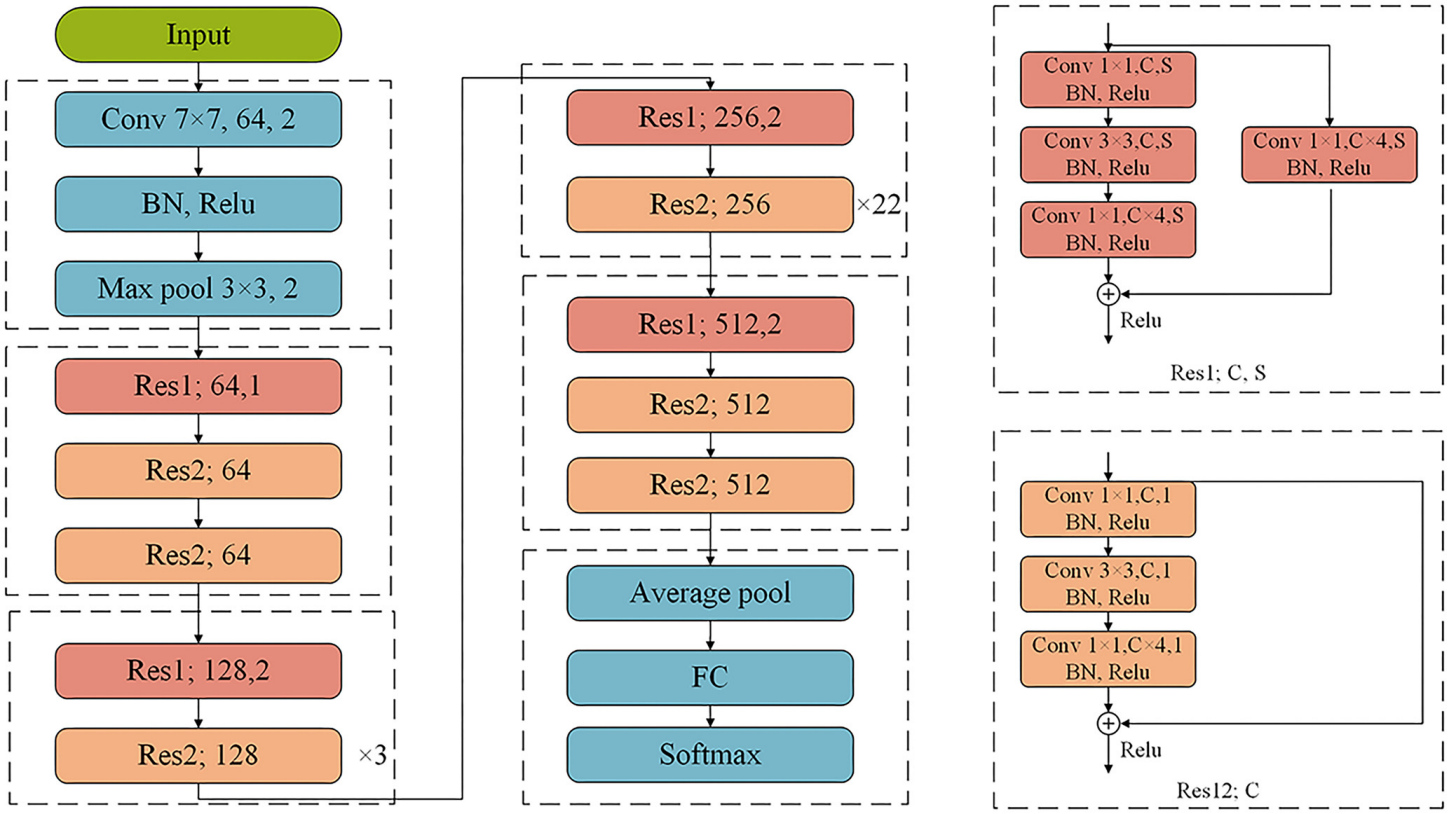
**ResNet101 architecture.** Res1 and Res2 stand for two types of residual block structures. C indicates the number of convolution kernels, and S represents the stride length.

**Figure 4.**
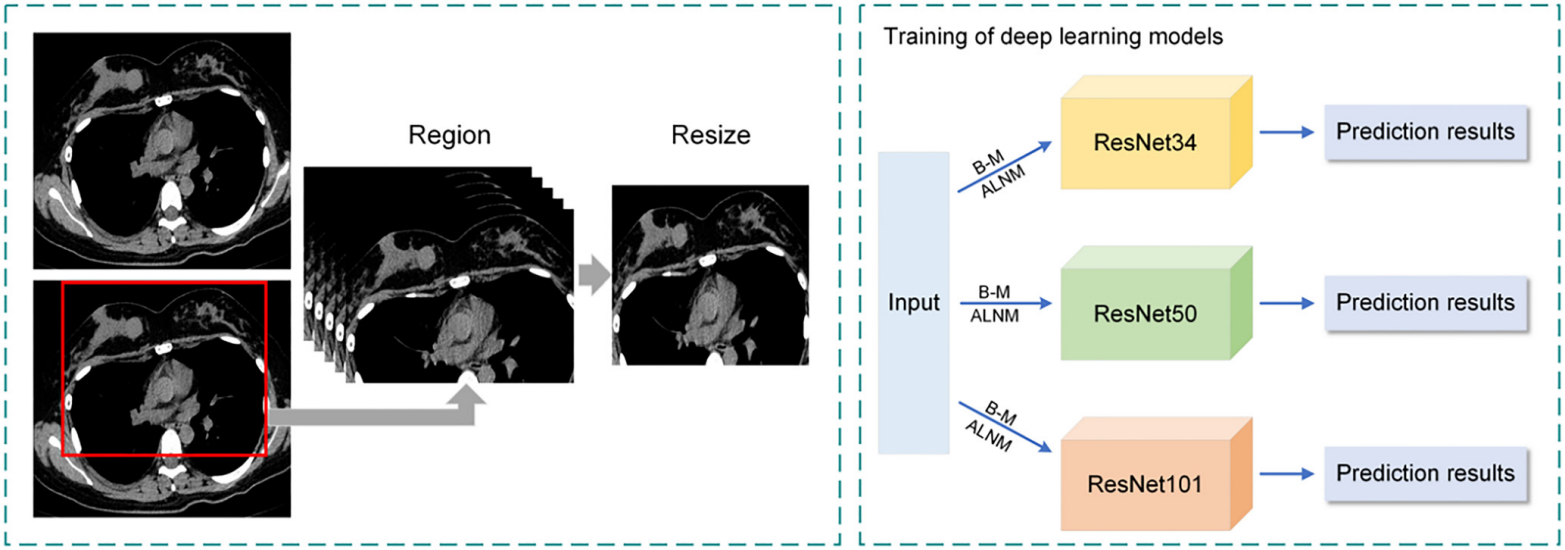
**Workflow and the basic structure of deep learning models.** B-M means the input of all chest CT images to identify benign and malignant breast masses; ALNM indicates the input of chest CT images of early breast cancer to predict axillary lymph node metastasis. Abbreviations: ALNM: Axillary lymph node metastasis; CT: Computed tomography.

The Residual Network (ResNet) is a deep CNN architecture widely used for breast tumor detection across various imaging modalities, including histopathological images, mammograms, MRI scans, US, and CT images [17–20]. Its strong generalization ability and robustness in handling variability in medical images make it a reliable choice. Different ResNet models can be configured by adjusting the number of channels and residual modules, such as ResNet18, ResNet34, ResNet50, ResNet101, and ResNet152. Figure 3 illustrates the ResNet101 architecture as an example. However, previous studies [21, 22] indicate that excessively increasing network depth can lead to ACC plateauing or even degrading, while also increasing computational costs. To balance classification ACC and operational efficiency, we developed prediction models based on ResNet34, ResNet50, and ResNet101 and evaluated their performance. The workflow for these models is shown in Figure 4. Our study utilizes chest CT images of breast masses at thin-layer mediastinal windows as input, with a feature classifier employing a Softmax scoring threshold of 0.5 for classification. The models were trained to predict benign and malignant breast masses as well as ALNM. The predictive models were trained and tested on a Windows-based image workstation using Python, the open-source deep learning library Torch, and an NVIDIA GeForce GTX 3080 Ti GPU. The detailed learning parameters for each model are presented in Table 3. Training was conducted over 100 epochs with a batch size of 16 samples per training set. The learning rate varied between 0.001 and 0.0001. After training, the algorithm was evaluated on a test set, producing probability scores ranging from 0 to 1 for early breast cancer detection and the presence of positive ALNs.

### Ethical statement

This retrospective study was approved by the First Affiliated Hospital of Shandong First Medical University (Shandong Provincial Qianfoshan Hospital), the Medical Ethics Committee (No. 2025-S026). The study adhered to local regulations and institutional requirements, and was conducted in accordance with the Declaration of Helsinki. The requirement for informed consent was waived due to the retrospective study design.

### Statistical analysis

All statistical analyses were performed using SPSS (version 26.0) and MedCalc (version 20.0). Normality and homogeneity of variance were assessed using the Shapiro–Wilk test and Levene’s test, respectively. Continuous variables following a normal distribution were presented as mean ± standard deviation (SD), while those with a skewed distribution were expressed as median and interquartile range (IQR). Categorical data were reported as frequencies. For group comparisons, independent-sample *t*-tests were used for normally distributed continuous variables, while Mann–Whitney *U* tests were applied to non-normally distributed data. Chi-square tests were conducted for categorical variables. A *P* value of <0.05 was considered statistically significant. The predictive model’s performance was evaluated using ACC, sensitivity (SEN), specificity (SPE), positive predictive value (PPV), and negative predictive value (NPV). Additionally, receiver operating characteristic (ROC) curve analysis and the area under the curve (AUC) were used to assess the model’s predictive efficacy.

## Results

The median age of all patients was 49 years (36.75–59). The malignant group had a median age of 57 years (48–67), while the benign group was significantly younger, with a median age of 37 years (26–49.75) (*P* < 0.05). However, tumor size did not differ significantly between the two groups. Within the malignant group, the ALNM-positive subgroup had a mean age of 55.99 ± 13.11 years and a mean tumor size of 2.52 ± 0.80 cm, whereas the ALNM-negative subgroup had a mean age of 57.69 ± 12.02 years and a mean tumor size of 2.43 ± 0.85 cm. No statistically significant differences were observed between these subgroups in age, tumor size, histological grade, or molecular subtype distribution (*P* > 0.05). These findings align with previous studies [16, 23, 24] and do not impact the validity of our deep learning experiments.

**Table 4:** Performance of the deep learning algorithm on chest CT in the test sets

	**ACC**	**SEN**	**SPE**	**PPV**	**NPV**	**AUC (95% CI)**
*Prediction model of benign and malignant breast masses*						
ResNet 34	0.842	0.945	0.879	0.906	0.928	0.962 (0.946–0.979)
ResNet 50	0.818	0.937	0.869	0.898	0.917	0.965 (0.952–0.979)
ResNet 101	0.859	0.929	0.917	0.933	0.913	0.964 (0.948–0.981)
*Prediction model of axillary lymph node metastasis*						
ResNet 34	0.760	0.730	0.733	0.596	0.834	0.819 (0.771–0.874)
ResNet 50	0.858	0.843	0.873	0.781	0.911	0.936 (0.909–0.966)
ResNet 101	0.874	0.865	0.909	0.837	0.926	0.951 (0.926–0.975)

Abbreviations: ACC: Accuracy; AUC: Area under the receiver operating characteristic curve; CI: Confidence interval; NPV: Negative predictive value; PPV: Positive predictive value; SEN: Sensitivity; SPE: Specificity.

**Figure 5.**
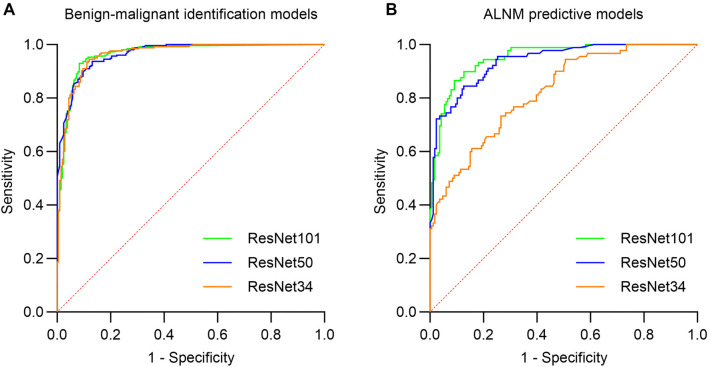
**The ROC curves of the model.** Abbreviations: ROC: Receiver operating characteristic; ALNM: Axillary lymph node metastasis.

The diagnostic efficiency metrics for each model are presented in Table 4. Among the models distinguishing between benign and malignant breast masses, the ResNet34 model achieved an AUC of 0.962 (95% CI: 0.946–0.979), with an ACC of 0.842, an SEN of 0.945, an SPE of 0.879, a PPV of 0.906, and an NPV of 0.928. The ResNet50 model showed similar performance, with an AUC of 0.965 (95% CI: 0.952–0.979), an ACC of 0.818, an SEN of 0.937, an SPE of 0.869, a PPV of 0.898, and an NPV of 0.917. The ResNet101 model performed comparably, achieving an AUC of 0.964 (95% CI: 0.948–0.981), an ACC of 0.859, an SEN of 0.929, an SPE of 0.917, a PPV of 0.933, and an NPV of 0.913. While the ResNet50 and ResNet101 models had similar AUC and NPV values, a comprehensive assessment of overall ACC and efficacy indicated that the ResNet101 model exhibited superior classification efficiency. The ROC curves for these models are shown in Figure 5A. For predicting ALNM in early-stage breast cancer, the ResNet34 model achieved an AUC of 0.819 (95% CI: 0.771–0.874), with an ACC of 0.760, an SEN of 0.730, an SPE of 0.733, a PPV of 0.596, and an NPV of 0.834. The ResNet50 model demonstrated better performance, with an AUC of 0.936 (95% CI: 0.909–0.966), an ACC of 0.858, an SEN of 0.843, an SPE of 0.873, a PPV of 0.781, and an NPV of 0.911. The ResNet101 model excelled further, achieving an AUC of 0.951 (95% CI: 0.926–0.975), an ACC of 0.874, an SEN of 0.865, an SPE of 0.909, a PPV of 0.837, and an NPV of 0.926. The results indicate that prediction efficiency improved with the number of convolutional layers, as reflected in the ROC curves in Figure 5B. Overall, the models demonstrated stronger predictive performance in distinguishing benign from malignant breast masses than in predicting ALNM. Among the three models evaluated, ResNet101 consistently exhibited the best performance across both tasks.

## Discussion

In this study, we successfully developed deep learning models with various architectures based on chest CT images. Our results demonstrate that these models can predict the nature of breast masses and the presence or absence of ALNM, with the ResNet101 model exhibiting the best predictive performance. This finding underscores the potential of deep learning models to enhance radiologists’ ability to assess breast abnormalities. Numerous studies have focused on differentiating benign from malignant breast tumors [14, 25, 26]. Unlike middle and late-stage breast cancer, early-stage breast cancer often presents subtle imaging characteristics, making visual differentiation more challenging. However, with increasing public health awareness and the widespread adoption of routine physical examinations, the detection window for breast cancer has advanced. Consequently, early-stage breast cancer diagnoses have risen, while middle and late-stage diagnoses have declined, particularly in developed countries with well-established healthcare systems [27]. Given this trend, distinguishing early-stage breast cancer from benign breast masses is of significant clinical importance. Additionally, assessing ALNM in early-stage cases is crucial for guiding surgical decisions and postoperative management. Non-contrast chest CT, a standard diagnostic tool for hospitalized patients and a common physical examination method, is primarily used to evaluate lung, cardiac, and mediastinal conditions. However, breast lesions are often overlooked [14]. While mammography and US remain the most widely used breast cancer screening methods [18, 28], non-contrast chest CT offers several advantages. Unlike mammography, it is painless, does not require multiple patient positions, and thus improves patient compliance while reducing anxiety. Compared to breast US, it provides a broader imaging range, higher spatial resolution, and clearer visualization of tumors and peritumoral structures. Additionally, non-contrast chest CT emits lower radiation doses than enhanced CT and PET-CT scans, eliminates the need for contrast agents, and reduces potential side effects—making it a safer, non-invasive alternative. It is also faster, more convenient, and more affordable than breast MRI. In conclusion, non-contrast chest CT presents an effective and patient-friendly option for breast cancer screening and evaluation. In future clinical practice, deep learning models could analyze routine chest CT images to provide direct and accurate breast lesion assessments, streamlining the diagnostic process.

In chest CT imaging for breast cancer diagnosis, previous studies have demonstrated the efficacy of radiomics—an approach that extracts quantitative features from medical imaging—for predicting breast lesions. For instance, some studies have used radiomics with chest CT images to characterize breast masses. Caballo et al. [29] systematically quantified the morphological characteristics of breast masses, extracted tumor margin features, and employed a linear discriminant analysis (LDA) classifier to develop a radiomic model for distinguishing between benign and malignant masses. Their model achieved an AUC of 0.90, demonstrating high diagnostic efficacy. However, this model was based on breast-specific CT (bCT) images, which require specialized equipment that is less accessible and convenient than conventional chest CT. Additionally, radiomic analysis has been effectively used to predict ALNM. Tang et al. [30] and Yang et al. [31] extracted radiomic features from CECT images and developed machine learning models to predict ALNM in breast cancer. Their models achieved accuracies of 0.83 and 0.89, with AUC values of 0.91 and 0.94, respectively. While both studies demonstrated strong experimental designs and excellent diagnostic performance, they relied on CECT images, which, despite enhancing breast cancer visualization, require contrast agent injection—posing a risk of adverse reactions. Overall, these studies indicate that CT imaging provides valuable diagnostic information for breast lesions and should not be overlooked. In recent years, a limited number of studies have developed deep learning models for the preoperative prediction of benign and malignant breast masses, as well as ALNM. Yasaka et al. [32] created a deep learning model based on contrast-enhanced chest CT images, achieving an AUC of 0.967—demonstrating its potential to assist radiologists in breast cancer detection. Similarly, Liu et al. [33] developed deep CNN models to predict ALN metastasis using CECT images. Their best-performing model, DA-VGG 19, achieved an ACC of 0.9088. While these models show high AUC and ACC, they primarily rely on CECT images and often address only a single prediction task, with limited exploration of multi-task models. Additionally, some studies have demonstrated the diagnostic value of non-contrast chest CT [34]. Given these findings, deep learning analysis of chest CT images—including non-contrast scans—holds promise for the preoperative assessment of both breast masses and ALN involvement in breast cancer.

In the present study, the subjects consisted of patients with benign masses and early-stage breast cancer rather than a natural dataset, leading to skewed age and tumor size distributions across all participants. The deep learning models developed based on chest CT images demonstrated improved ACC in distinguishing between benign and malignant breast masses and predicting ALNM. These findings align with the results reported by Yasaka et al. [32] and Liu et al. [33]. In both prediction tasks, the performance of the three models showed a gradual improvement as the number of layers increased, consistent with He et al.’s [21] research on the relationship between network depth and ACC in ResNet. Furthermore, a comparison of the two tasks revealed that distinguishing between benign and malignant breast masses (task a) outperformed the prediction of ALNM (task b). This discrepancy may stem from the fact that breast mass features—such as shape, edge, and density—are typically more distinct and, therefore, easier for deep learning models to capture [26, 32]. In contrast, ALNM prediction may involve more complex and multifactorial characteristics [15]. Additionally, our study focused exclusively on early-stage breast cancer for ALNM prediction, where malignant traits were less pronounced, potentially affecting the predictive results. Moving forward, expanding our dataset to include a broader range of cases will be essential. Moreover, we aim to develop models specifically optimized for ALNM prediction to further enhance performance. This study leveraged breast imaging information from non-contrast chest CT scans, offering a novel approach to breast lesion screening. Since chest CT is commonly performed in routine health checkups, integrating breast lesion assessment into this process could facilitate the clinical application of our models. Our study has some limitations. First, it is a retrospective analysis that lacks multi-center data, necessitating future multi-center external validation and prospective studies to confirm these findings. Second, the malignant group in our dataset consisted solely of early-stage breast cancer cases, and the dataset used for ALNM prediction was imbalanced, underscoring the need for more comprehensive datasets in future research. Third, the CT images were obtained from various scanners, introducing potential noise that could impact model ACC. However, given the principles of multi-center validation, we believe the models developed in this study are relatively robust and likely to yield similar results in other medical centers.

## Conclusion

In conclusion, our findings suggest that deep learning models using non-contrast chest CT images can provide clinicians with valuable preoperative insights into breast masses and ALNs during physical examinations or initial hospital admissions. This, in turn, supports clinical decision making. Our study highlights the convergence of conventional imaging techniques and advanced AI technology, offering a more cost-effective and efficient approach to predicting breast lesions.

## Data Availability

The raw data supporting the conclusions of this article will be made available by the corresponding author, without undue reservation.
